# A Role of Histone Acetylation in the Regulation of Circadian Rhythm in Ants

**DOI:** 10.1016/j.isci.2020.100846

**Published:** 2020-01-17

**Authors:** Romain Libbrecht, Dennis Nadrau, Susanne Foitzik

**Affiliations:** 1 Institute of Organismic and Molecular Evolution (IOME), Johannes Gutenberg University Mainz, Biozentrum I, Hanns Dieter Hüsch Weg 15, 55128 Mainz, Germany

**Keywords:** Entomology, Molecular Biology, Molecular Mechanism of Behavior

## Abstract

In many organisms, circadian rhythms and associated oscillations in gene expression are controlled by post-translational modifications of histone proteins. Although epigenetic mechanisms influence key aspects of insect societies, their implication in regulating circadian rhythms has not been studied in social insects. Here we ask whether histone acetylation plays a role in adjusting circadian activity in the ant *Temnothorax longispinosus*. We characterized activity patterns in 20 colonies to reveal that these ants exhibit a diurnal rhythm in colony-level activity and can rapidly respond to changes in the light regime. Then we fed *T. longispinosus* colonies with C646, a chemical inhibitor of histone acetyltransferases, to show that treated colonies lost their circadian rhythmicity and failed to adjust their activity to the light regime. These findings suggest a role for histone acetylation in controlling rhythmicity in ants and implicate epigenetic processes in the regulation of circadian rhythms in a social context.

## Highlights

• Colony-level activity shows diurnal rhythmicity in *Temnothorax longispinosus*
• *T. longispinosus* colonies adjust their activity in response to a new light regime
• Colonies treated with an inhibitor of histone acetylation lose their rhythmicity
• Our findings suggest an epigenetic regulation of circadian rhythms in ants

## Graphical Abstract

**Figure:**
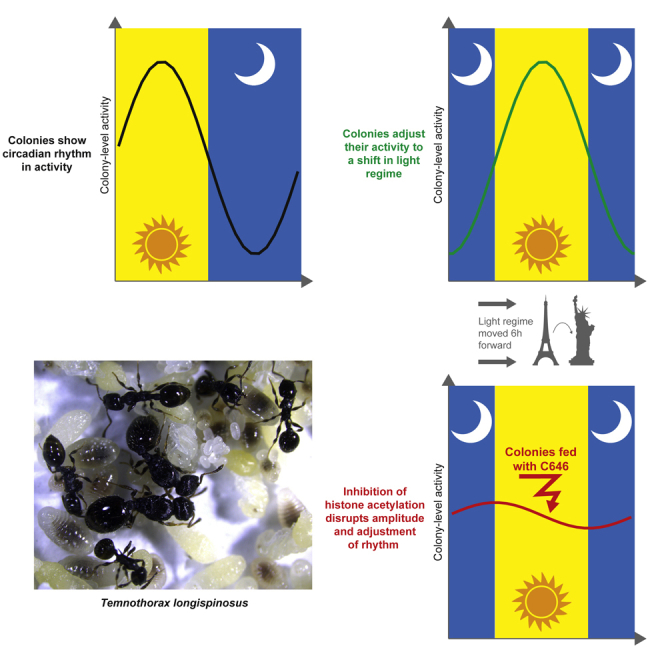

## Introduction

The rotation of the earth around the sun causes daily rhythmicity in environmental conditions. Living organisms respond to these predictable fluctuations by expressing daily rhythms in physiology, metabolism, and activity (Hut and Beersma, 2011). Circadian rhythms have been described across the Tree of Life and at multiple scales, from unicellular organisms to animal societies (Bell-Pedersen et al., 2005). Although most animal cells can maintain rhythmic processes and metabolic activities, the synchronization between the organisms' rhythm and external cues (e.g., light) is typically controlled and maintained by the circadian clock, which acts as a central pacemaker in the brain (Mendoza-Viveros et al., 2017, Takahashi, 2017).

The circadian clock is a set of conserved proteins that interact to regulate daily oscillations in gene expression and protein production (Mendoza-Viveros et al., 2017). The molecular regulation of circadian rhythms is conserved across the animal kingdom, although the specific role and nature of different components of this molecular clock varies across species (Bell-Pedersen et al., 2005, Young and Kay, 2001). The CLOCK protein is central to the molecular clock, as it forms a heterodimer with BMAL1 (in mammals) or CYCLE (in flies) and acts as a transcription factor that drives downstream transcriptional changes by binding to enhancer-boxes (E-boxes) in the promoter region of other clock genes (Bell-Pedersen et al., 2005). Molecular clock activity results in large-scale downstream changes in gene expression (Zhang et al., 2014) that may involve less targeted gene regulatory routes (Takahashi, 2017).

The molecular clock regulates rhythmic oscillations in gene expression via several epigenetic processes, including histone modifications. DNA wraps around histone proteins and any alterations of these histones can affect the ability of other proteins (e.g., transcription factors) to bind to regulatory regions of the genome and thus influence transcription (Huang et al., 2014, Lawrence et al., 2016). Rhythmic changes in histone modifications could thus enable oscillating patterns of gene expression. Clock proteins can affect the acetylation status of the N-terminal tail of histones. In mammals, the CLOCK-BMAL1 complex associates with histone acetyltransferases of the p300 and CREB-binding protein (CBP) families (Etchegaray et al., 2003, Hosoda et al., 2009). Furthermore, the mammalian CLOCK shares similarity with histone acetyltransferases of the MYST and ACTR families (Doi et al., 2006) and has intrinsic histone acetyltransferase properties that are enhanced when associated with BMAL1. In *Drosophila*, CBP histone acetyltransferases regulate the circadian rhythm by modulating the transcription of *clock* and *cycle* and thus the production of the CLOCK/CYCLE heterodimer (Lim et al., 2007). Other epigenetic processes underlying rhythmic oscillations of gene expression include histone methylation (Zheng et al., 2018), DNA methylation (Azzi et al., 2014), and regulation via non-coding RNAs (Bhadra et al., 2018).

Although daily rhythmicity and its molecular basis are conserved across animals, intraspecific variation in circadian rhythms can be important and explained by both intrinsic and extrinsic parameters (van der Veen et al., 2017). In particular, circadian rhythms of social insects are influenced by task specialization and social context. Ant and bee workers that forage for food typically show rhythmic activity, whereas workers that nurse the brood inside the nest are often arrhythmic, fulfilling their social tasks around the clock (Fujioka et al., 2017, Ingram et al., 2009, Mildner and Roces, 2017, Moore et al., 1998, Sharma et al., 2004, Shemesh et al., 2010, Shemesh et al., 2007; but see Fuchikawa et al., 2014). Previous studies revealed that the presence of brood reduces circadian fluctuations in the activity of honeybee and ant nurse workers (Fujioka et al., 2017, Shemesh et al., 2010) and bumblebee founding queens (Eban-Rothschild et al., 2011), possibly because larvae need constant care. This indicates that the social environment affects circadian rhythms in social Hymenoptera (Fujioka et al., 2019). However, ants and bees can maintain circadian rhythmicity in complete darkness (Bloch et al., 2001, Sharma et al., 2004) and can re-synchronize their circadian rhythm upon changes in the timing of the light regime (Mildner and Roces, 2017). Interestingly, most studies on the circadian rhythm of social insects measured individual activity or used individual activity to estimate the proportion of rhythmic individuals in a group. Although some studies approached the issue at the colony level (e.g., rhythmicity of oxygen consumption by groups of honeybees (Moritz and Kryger, 1994)), there is a gap in our understanding of group-level circadian rhythm in insect societies.

Circadian activity in social insects is also controlled by the molecular clock and is associated with oscillating patterns of clock gene expression. Surprisingly, the molecular clock machinery in honeybees and fire ants is more similar to the mammalian clock than to the *Drosophila* clock in term of gene presence, sequence similarity, binding domains, and patterns of gene expression (Ingram et al., 2012, Weinstock et al., 2006). The expression of clock genes shows daily oscillations in bees (Rodriguez-Zas et al., 2012, Shemesh et al., 2010, Shemesh et al., 2007, Toma et al., 2000) and ants (Ingram et al., 2012, Ingram et al., 2009) for rhythmic individuals, but no or weaker oscillations for arrhythmic nurses that care for the brood (Ingram et al., 2009, Rodriguez-Zas et al., 2012, Shemesh et al., 2010, Shemesh et al., 2007), although levels of the clock protein PERIOD do oscillate over the course of the day in arrhythmic bee nurses (Fuchikawa et al., 2017). The behavioral rhythmicity observed in social insects suggests daily fluctuations in the expression of many genes (i.e., not restricted to clock genes), but there is no study of the gene regulatory mechanisms modulating such oscillating patterns in gene expression.

In this experimental study, we first investigated whether workers of the ant species *Temnothorax longispinosus* show group-level circadian rhythmicity in the proportion of foragers and the proportion of active ants inside the nest. Second, we raised the question whether histone acetylation regulates gene expression changes underlying rhythmic activity in ants. We predicted that, as histone modifications regulate circadian rhythm in other animals (including flies), inhibiting the activity of p300/CBP histone acetyltransferases may reduce behavioral rhythmicity in *T. longispinosus*. Third, we shifted the daily light-darkness cycle 6 h forward to observe whether ants adapt their activity rhythm to this change and to test whether inhibition of histone acetylation altered this response.

## Results

**Figure 1:**
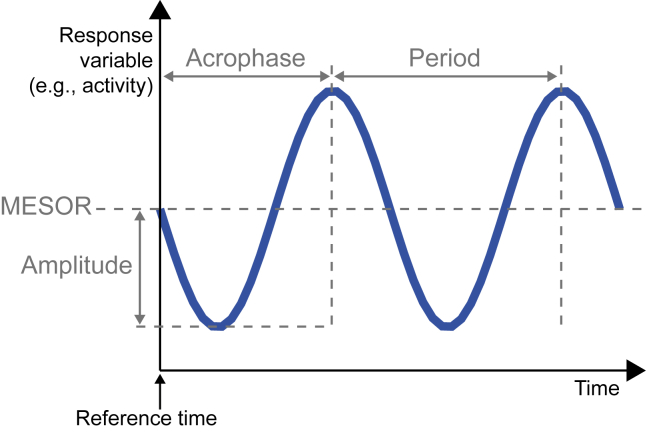
Cosinor-Based Analyses of Rhythmicity Estimate Four Parameters of the Rhythm The MESOR (Midline Estimating Statistics of Rhythm) is the average estimated value. The amplitude is half the extent of the change in estimated value. The acrophase is the timing of the first peak relative to the reference time. The period is the duration of one cycle. Adapted from Cornelissen (2014).

To characterize the behavioral rhythmicity of *T. longispinosus* colonies, 20 laboratory nests were filmed continuously for four days under a 12 h:12 h light:dark regime. Every hour, we recorded the proportion of active ants inside the nest and the proportion of ants foraging outside the nest. To test whether these colony-level measurements followed a circadian rhythm, we performed cosinor-based analyses of rhythmicity (see Transparent Methods for details), which also calculated parameters of the cosine function fitted to the behavioral changes over time (Cornelissen, 2014) (Figure 1).

### Activity Inside the Nest and Foraging Show Rhythmicity

**Figure 2:**
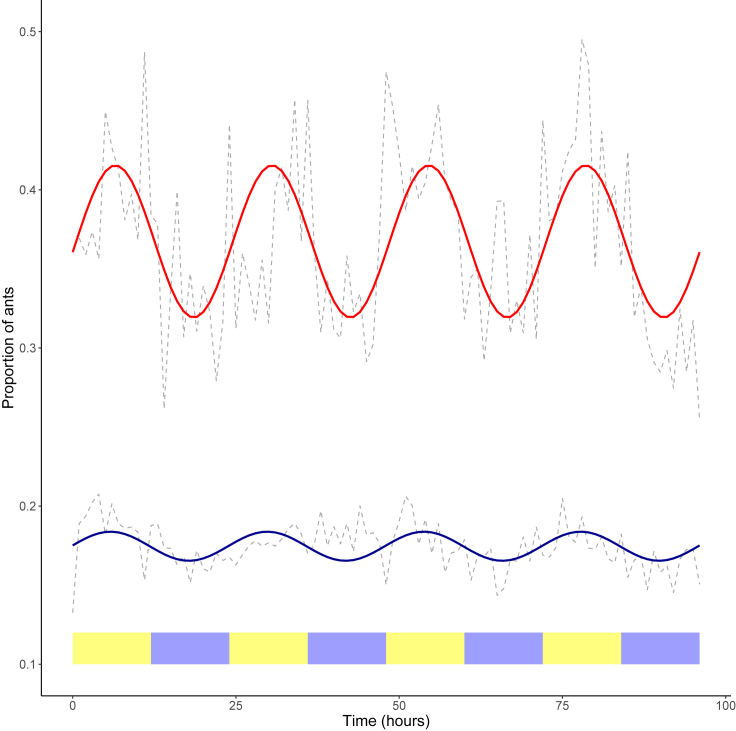
Proportion of Active Ants inside the Nest (in Red) and Proportion of Ants Foraging outside (in Blue) Gray dotted line = empirical data (mean of the 20 colonies). Red solid line = expected data (cosine function that best summarizes the empirical data). Yellow boxes indicate light periods, and blue boxes indicate dark periods.

The cosine function that best summarized the changes over time in the activity inside the nest was characterized by an MESOR parameter of 0.37 (confidence interval: 0.32–0.41), an amplitude of 0.048 (confidence interval: 0.034–0.062), and an acrophase of 6.53 h (confidence interval: 5.46–7.52) (Figure 2). The finding that zero is not within the confidence interval of the amplitude reveals that the proportion of active ants inside the nest follows a circadian rhythm. This rhythmicity was also confirmed by a rhythm detection test (F_2,18_ = 23.7, p < 0.0001) (Bingham et al., 1982).

The cosine function summarizing changes over time in the proportion of ants outside the nest yielded an MESOR parameter of 0.17 (confidence interval: 0.15–0.20), an amplitude of 0.0092 (confidence interval: 0.0048–0.014), and an acrophase of 5.81 h (confidence interval: 3.63–8.33) (Figure 2). Again, the finding that zero is below the confidence interval of the amplitude shows that the proportion of ants outside the nest follows a circadian rhythm, which was confirmed by a rhythm detection test (F_2,18_ = 9.31, p = 0.0017) (Bingham et al., 1982). The rhythm explained less variance in the proportion of foragers (19.4%) than in the proportion of active ants inside (35.3%), and the MESOR differed significantly between the two measurements (F_1,38_ = 70.17, p < 0.0001).

### Characterization of the Circadian Rhythm

The circadian rhythm of the proportion of active ants inside the nest showed an acrophase of 6.39 ± 2.66 h, which did not differ significantly from the middle of the light period (t test vs 6, t = 0.64, df = 18, p = 0.53). Inside ants were thus more active during the day, than during the night. Similarly, the acrophase for the proportion of foragers was 6.57 ± 3.94 h, which did not differ significantly from the middle of the light period (t test vs 6, t = 0.63, df = 18, p = 0.53).

### C646 Treatment Affects Behavioral Rhythms

To investigate a potential role of histone acetylation in regulating the circadian rhythm described in *T. longispinosus*, we supplemented the food of the ant colonies with either a control solution or a solution containing C646, a selective inhibitor of p300/CBP histone acetyltransferases (Bowers et al., 2010). After 20 days of treatment, we shifted the light regime six hours forward before filming all colonies continuously to record hourly proportions of foragers and active ants inside the nest.

#### Activity inside the Nest

**Figure 3:**
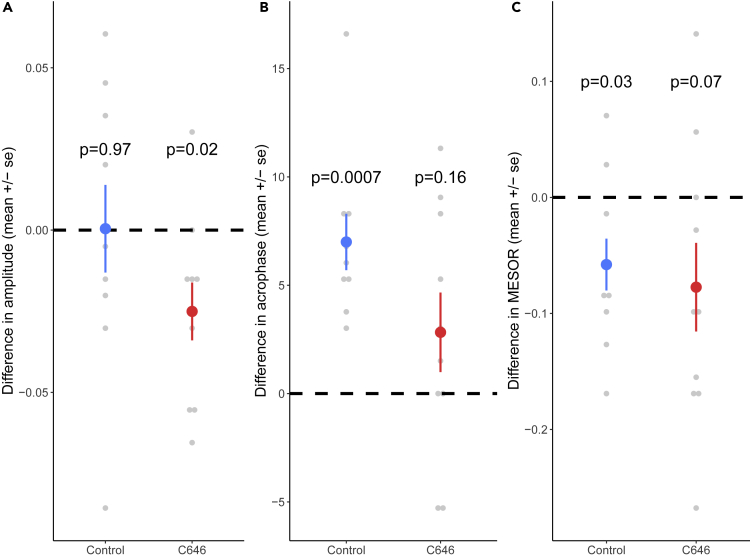
The Effect of the Light Change on the Rhythmicity of the Activity of Inside Workers Differed between Control and C646 Treatments (A) The amplitude of the rhythm was decreased in the C646 (n = 10)—but not the control (n = 10)—colonies. (B) The acrophase moved forward for the control (n = 9)—but not the C646 (n = 10)—colonies. (C) There was a weak reduction of the MESOR after the light change for both control (n = 10) and C646 (n = 10) colonies. Reported p values correspond to the comparison to zero.

We found that although the control colonies kept a significant rhythmicity for the activity inside the nest after the light change (F_2,8_ = 5.34, p = 0.034), the C646-treated colonies did not show such rhythmicity anymore (F_2,8_ = 1.71, p = 0.24). To better characterize the effect of C646 on circadian rhythm and take into account that the same colonies were tested before and after the light change, we extracted values for the parameters of the rhythm before and after the light change and calculated for each colony the difference in amplitude, acrophase, and MESOR between before and after the light change. This confirmed our finding that C646 affected the rhythmicity, as the amplitude was significantly reduced for the C646 treatment (t test vs 0, t = −2.81, df = 9, p = 0.02) but not for the control colonies (t test vs 0, t = 0.032, df = 9, p = 0.97, Figure 3A). Our analysis suggested that control colonies shifted their daily rhythms to the new light regime, whereas colonies treated with C646 did not. We found that the shift in acrophase for control colonies (6.99 ± 3.90 h) differed significantly from zero hour (t test vs 0, t = 5.37, df = 8, p = 0.0007), whereas it did not in the C646 treatment (2.83 ± 8.81 h; t test vs 0, t = 1.54, df = 9, p = 0.16, Figure 3B). Finally the MESOR—and therefore the average activity—showed a weak reduction after the light change for both the control (t test vs 0, t = −2.59, df = 9, p = 0.03) and the C646 colonies (t test vs 0, t = −2.03, df = 9, p = 0.07, Figure 3C).

#### Foraging

**Figure 4:**
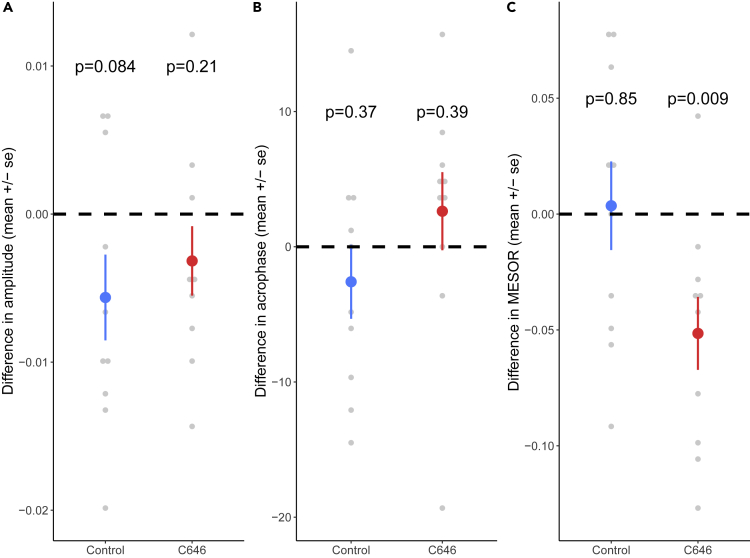
Effect of the Light Change on Characteristics of the Rhythmicity of the Proportion of Foragers (A) The light change did not affect the amplitude for both control (n = 10) and C646 (n = 10) colonies. (B) The light change did not affect the acrophase for both control (n = 10) and C646 (n = 10) colonies. (C) The MESOR was reduced after the light change for C646 (n = 10) colonies but not for control (n = 10) colonies. Reported p values correspond to the comparison to zero.

After the light change, the control colonies were still characterized by a significant rhythmicity (F_2,8_ = 6.87, p = 0.02), contrary to the colonies treated with C646 (F_2,8_ = 2.72, p = 0.13). However, the amplitude of the rhythm was not significantly reduced for the control (t test vs 0, t = −1.94, df = 9, p = 0.084) and the C646 colonies (t test vs 0, t = −1.35, df = 9, p = 0.21; Figure 4A). We also found that the difference in acrophase did not differ from zero hour both for control (−2.59 ± 8.65 h, t test vs 0, t = −0.95, df = 9, p = 0.37) and C646-treated colonies (2.63 ± 9.13 h, t test vs 0, t = 0.91, df = 9, p = 0.39; Figure 4B). Finally, the MESOR showed a reduction in foraging activity after the light change for the C646 colonies (t test vs 0, t = −3.27, df = 9, p = 0.009) but not for the control colonies (t test vs 0, t = 0.19, df = 9, p = 0.85, Figure 4C).

## Discussion

In this experimental study, we used continuous video recordings of laboratory colonies of the ant *Temnothorax longispinosus* to describe daily rhythms in colony-level activity. The ant colonies showed a clear daily rhythm with peak activity around the middle of the day, and this rhythmicity was more pronounced for the activity inside the nest compared with the foraging activity. Second, we tested the role of histone acetylation in regulating this circadian rhythm by treating the ant colonies with C646, an inhibitor of p300/CBP histone acetyltransferases. The C646 treatment altered the daily activity and prevented its adjustment to a new light:dark rhythm, suggesting that histone acetylation is an essential mechanism for both maintaining and entraining colony-level circadian activity in social insects. Finally, the foraging activity was decreased under inhibition of p300/CBP histone acetyltransferases, which is consistent with a role of histone acetylation in the regulation of foraging, as reported in another ant species (Simola et al., 2016).

The finding that colonies treated with C646 lost their rhythmicity in intranidal activity—and to a lesser extent in foraging activity—is consistent with an epigenetic regulation of circadian rhythm in *T. longispinosus*. More specifically, it suggests that histone acetyltransferases of the p300/CBP family—and thus the acetylation of histone proteins—are involved in maintaining the circadian rhythm. This is consistent with findings in mammals and insects of a similar role of p300/CBP histone acetyltransferases in regulating circadian activity. In *Drosophila*, CBPs downregulate the transcription of the CLOCK/CYCLE heterodimer (Lim et al., 2007). In mammals, the clock proteins CLOCK and BMAL1 interact with p300/CBP histone acetyltransferases to acetylate histones and thus regulate circadian fluctuations in transcription (Etchegaray et al., 2003, Hosoda et al., 2009). In addition, the mammalian CLOCK itself has some histone acetyltransferase activity (Doi et al., 2006). All this evidence indicates a conserved role for histone acetylation in regulating circadian rhythms in animals, which is further supported by our findings.

In addition to decreasing the strength of the rhythm, the C646 treatment also prevented the ants from moving their in-nest activity forward in response to a shift in the light:dark regime. There was no such effect of C646 for the foraging activity, because we failed to detect any shift in rhythmicity for this measure, including in control colonies, possibly due to its weaker amplitude. However, the effect of C646 on the ability of in-nest ants to adjust their activity suggests that histone acetylation—and more generally, epigenetic processes—are involved in the entrainment of the circadian rhythm to external cues. Alternatively, this finding could be a mere consequence of the loss in rhythmicity triggered by the treatment, which prevented the ants from shifting their activity forward or us from detecting such a shift. Our results call for more studies on the epigenetic adjustment of circadian rhythm in response to external changes.

Interestingly, we detected a stronger circadian rhythm for the activity inside the nest than for the foraging activity. This stands in contrast to previous studies of circadian rhythm in social insects that reported context-specific strength of circadian rhythm. Specifically, most studies could detect a circadian rhythm for foraging activity but failed to do so for brood care behavior (Mildner and Roces, 2017, Moore et al., 1998, Shemesh et al., 2007). A possible explanation put forward was that larvae need care around-the-clock, whereas foraging efficiency varies depending on the time of the day. This hypothesis was supported by the finding that experimental addition of brood to rhythmic individuals dramatically decreases the strength of their rhythm in *Diacamma* ants (Fujioka et al., 2017; but see Fuchikawa et al., 2014), bumblebees (Eban-Rothschild et al., 2011), and honeybees (Shemesh et al., 2010). One specificity of our study is that we recorded any type of activity inside the nest that was not limited to nursing behavior. Thus, what we detected as in-nest rhythmicity did not only reflect rhythmicity in nursing behavior but could also stem from rhythmicity in locomotor activity. In addition, all ants—both inside and outside—had access to light. This situation is not unnatural for *Temnothorax* ants (Foitzik et al., 2004), as they inhabit small ephemeral nest sites such as acorns, where light shines in through the nest entrance. In addition, previous studies that reported an absence of rhythm for ant nurse workers also provided them with access to light (Fujioka et al., 2017, Mildner and Roces, 2017, Sharma et al., 2004). However, bee nurses show stronger synchronization when exposed to a light:dark regime (Fuchikawa et al., 2016, Shemesh et al., 2007, Shemesh et al., 2010), which could facilitate the detection of group-level rhythmicity. Therefore, we cannot rule out that the access to light explains discrepancies between studies, and it remains to be understood whether and why task- or location-specific circadian rhythmicity varies across species.

We also report the detection of a circadian rhythm at the group level (rhythmicity in the proportion of individuals with a specific behavior) that is not based on the detection of individual rhythmicity. Previous social insect studies typically reported circadian rhythm at the individual level (rhythmicity in individual behavioral performance) or indirectly at the group level (using the proportion of rhythmic and arrhythmic individuals) (Eban-Rothschild et al., 2011, Fujioka et al., 2017, Mildner and Roces, 2017, Moore et al., 1998, Rodriguez-Zas et al., 2012, Sharma et al., 2004). Our finding raises questions on the relationship between different levels of rhythmicity, for example as to whether group-level rhythmicity necessarily requires rhythmicity at the individual level. Future studies on this issue will complement recent efforts to investigate whether individual rhythmicity is influenced by the rhythmicity of other group members (Fujioka et al., 2019). More generally, understanding circadian rhythm in a group or a society will require testing individual and group-level rhythmicity, as well as how one affects the other. In addition, it would be important to investigate whether some individuals are central to the group-level rhythmicity, as is the case for some cells playing the role of central peacemaker within a single multicellular organism (Mendoza-Viveros et al., 2017, Takahashi, 2017). Although our study does not provide such information across different phenotypic levels, it raises interesting questions by revealing the existence of circadian rhythmicity at the colony level in *Temnothorax* ants.

Inhibition of the p300/CBP histone acetyltransferases was expected to affect not only the circadian rhythmicity but other behaviors as well. Indeed, we demonstrated that colonies treated with C646 showed reduced foraging activity, irrespective of any rhythmic patterns. Although we cannot rule out light toxicity effects of the C646 treatment (see Transparent Methods), this finding is consistent with a recent study in *Camponotus* carpenter ants, which describes decreased scouting and foraging activity in colonies fed with similar C646 treatments as in our study (Simola et al., 2016). This suggests a conserved epigenetic regulation of foraging in insects (Anreiter et al., 2017) and more generally of behavior in animals (Dias et al., 2015, Marek et al., 2011).

To conclude, this study implicates histone acetylation in the regulation and entrainment of circadian rhythm in ants. Future studies should confirm these findings in other social insects and contrast the mechanistic regulation of rhythmicity across phenotypic levels (individual and colony level) to better understand circadian rhythm in a social context. Importantly, our study adds to the growing body of evidence for the implications of epigenetic processes in regulating social life in insects (Herb et al., 2012, Kucharski et al., 2008, Libbrecht et al., 2013, Libbrecht et al., 2016, Marshall et al., 2019, Morandin et al., 2019, Simola et al., 2016) and more generally circadian rhythm in animals (Mendoza-Viveros et al., 2017, Stevenson, 2018).

### Limitations of the Study

As discussed above, intranidal workers were directly exposed to light. Although the small nest sites that characterize this species usually allow some light to reach in-nest workers under natural conditions (Foitzik et al., 2004), it is unlikely that the entire nest is as well lit as in our experimental set-up. In all the experiments, the ants were exposed to the light:dark regime, thus we cannot rule out that the observed rhythmicity stems from a direct response to light, rather than an internal regulation. Another limitation is that our study does not quantify the molecular effects of C646, thus despite a successful decrease in histone acetylation after feeding similar concentrations of C646 in another ant species (Simola et al., 2016), we cannot be entirely sure that the phenotypic effects are triggered by the molecular changes of histones. Similarly, our study does not resolve specific molecular changes underlying circadian rhythmicity. However, we provide support for an epigenetic regulation of circadian rhythm in insect societies, and future studies can build on our findings to further characterize the molecular regulation of circadian rhythm in social insects.

## Methods

All methods can be found in the accompanying Transparent Methods supplemental file.

## Data and Code Availability

The data is available here: https://doi.org/10.17632/b23zr3tj39.1.

## Supplemental Information

Document S1. Transparent Methods
