# Supplementary material for: A Role of Histone Acetylation in the Regulation of Circadian Rhythm in Ants
Source: iScience. 2020 Jan 17;23(2):100846. doi: 10.1016/j.isci.2020.100846 (PMC6995257; doi:10.1016/j.isci.2020.100846)
Supplement: Document S1. Transparent Methods [file mmc1.pdf]

iScience, Volume *23*

Supplemental Information

A Role of Histone Acetylation in the Regulation of Circadian Rhythm in Ants

Romain Libbrecht, Dennis Nadrau, and Susanne Foitzik

Transparent Methods

Study system

Colonies of the ant *T. longispinosus* were collected in spring 2016 at the E. N. Huyck Preserve, in Rensselearville (New York, USA). A collection permit was issued by the Preserve. We transported the ant colonies in sealed plastic bags with some nesting material to the Johannes Gutenberg University of Mainz. Each of the 20 colonies used in this study contained one queen, at least 15 workers, as well as brood of all stages including eggs, larvae and pupae. Throughout the experiments, ants were housed in artificial nests composed of two microscope glass slides separated by a pre-cut Plexiglas (50x10x3mm) placed in three-chambered plastered plastic boxes, kept at 25°C ± 1°C under a 12h:12h light:dark regime, provided with water *ad libitum*, and fed with honey and crickets twice a week.

Behavioral observations

To characterize the behavioral rhythmicity, nests were individually transferred to a foraging arena (300x225x50mm), and videotaped continuously for four days using Canon Legria HF R706 cameras mounted on tripods (the distance between the camera lens and the nest was app. 5cm). To allow continuous filming, memory cards were replaced every day at 8am (beginning of the light phase) and 8pm (end of the light phase). The dark phase was simulated using red light that produced wavelengths (>620nm) outside of the detection range of the ants (Briscoe and Chittka, 2001). The videos were then used to score the position (inside or outside the nest) and behavior (walking, feeding, grooming, antennating, resting) of all ants once every hour for 96 hours (four days). We recorded the proportion of ants inside the nest that were active (i.e., not resting), as well as the proportion of ants outside the nest. This experimental design limited the number of colonies that we could videotape simultaneously, thus the colonies were processed in four cohorts of five colonies.

Detection and characterization of behavioral rhythmicity

To test whether behavioral changes over time showed a 24-hour rhythmicity (circadian rhythm), we used the command population.cosinor.lm of the cosinor2 R package. This command provides a rhythm detection test for a period of 24 hours, as well as the parameters (and their confidence intervals) of the cosine function that best summarizes the behavioral changes over time, when all 20 colonies are considered. The first parameter is MESOR (Midline Estimating Statistic Of Rhythm), which corresponds to the average rhythm-adjusted estimated value (Cornelissen, 2014). The second parameter is the amplitude, which corresponds to half the extent of change within a cycle. The third parameter is the acrophase, which corresponds to the timing (in hours) of the first peak relative to the reference time (Cornelissen, 2014) (Figure 1).

In addition to this population-level approach, we used the command cosinor.lm of the cosinor2 R package to extract for each colony the parameters of the cosine function that best summarizes the behavioral changes over time, thus allowing us to estimate variation among colonies. For each parameter, the values for all 20 colonies were checked for outliers by visual inspection of the distribution of the residuals. One outlier was removed for the acrophase of the proportion of active ants inside the nest (colony NY16F881), the acrophase of the proportion of ants outside the nest (colony NY16F422), and the MESOR of the proportion of ants outside the nest (colony NY16O525).

Inhibition of p300/CBP histone acetyltransferases

After the 20 colonies were videotaped for four days, they were randomly assigned to a control (n = 10) or a treatment (n = 10) group. Colonies in the treatment group were fed daily for 20 days with 15µl of 100 µM C646 (Absource Diagnostics) and 100 µM DMSO (Carl Roth) dissolved in 0.102 g/ml sucrose solution, whereby control colonies were fed with 15µl of 100

μM DMSO dissolved in 0.102 g/ml sucrose solution. C646 selectively inhibits the activity of p300/CBP histone acetyltransferases, a family of enzymes that acetylate histones (Bowers et al., 2010). Feeding such concentration of C646 decreased histone acetylation (H3K27ac) and affected gene expression in the brain in another ant species (Simola et al., 2016). We could not detect any effect of the C646 treatment on the difference in the number of ants between after and before the treatment (Control = 3.9 ± 4.2 (mean ± sd), C646 = 3.8 ± 7.8; t-test: $t = -0.036$, $p = 0.97$). Although this is not consistent with lethal toxicity, we cannot completely rule out some light toxicity of the C646 treatment.

Shift of light regime and the effect on behavioral rhythmicity

After the 20 colonies received the treatment for 20 days, we shifted the light regime six hours forward (light phase from 2pm to 2am, dark phase from 2am to 2pm). The next day at 2pm, we started the recording of all colonies to score the proportion of active ants inside the nest and the proportion of ants outside the nest every hour for four days (as described above). Preliminary analyses revealed that the behavior of ants was strongly affected by manipulations in the first 24 hours and thus all comparisons of control and C646 colonies were conducted while excluding the first 24 hours of recording. To test whether control and C646-treated colonies responded differently to the light change, we calculated for each parameter the difference between after and before the treatment, and compared these values to 0 (i.e., no change) using one-sample t-tests for both the control and C646 treatments. We performed visual inspection of the distribution of the residuals to detect outliers, and removed one outlier for the difference in acrophase of the proportion of active ants inside the nest (colony NY16F881).

References

Bowers, E.M., Yan, G., Mukherjee, C., Orry, A., Wang, L., Holbert, M.A., Crump, N.T., Hazzalin, C.A., Liszczak, G., Yuan, H., Larocca, C., Saldanha, S.A., Abagyan, R., Sun, Y., Meyers, D.J., Marmorstein, R., Mahadevan, L.C., Alani, R.M., Cole, P.A., 2010. Virtual ligand screening of the p300/CBP histone acetyltransferase: identification of a selective small molecule inhibitor. Chem. Biol. 17, 471–482. https://doi.org/10.1016/j.chembiol.2010.03.006

Briscoe, A.D., Chittka, L., 2001. The evolution of color vision in insects. Annu. Rev. Entomol. 46, 471–510. https://doi.org/10.1146/annurev.ento.46.1.471

Cornelissen, G., 2014. Cosinor-based rhythmometry. Theor. Biol. Med. Model. 11, 16. https://doi.org/10.1186/1742-4682-11-16

Simola, D.F., Graham, R.J., Brady, C.M., Enzmann, B.L., Desplan, C., Ray, A., Zwiebel, L.J., Bonasio, R., Reinberg, D., Liebig, J., 2016. Epigenetic (re) programming of caste-specific behavior in the ant *Camponotus floridanus*. Science 351, aac6633.
